# A novel system for spatial and temporal imaging of intrinsic plant water use efficiency

**DOI:** 10.1093/jxb/ert288

**Published:** 2013-09-16

**Authors:** L. McAusland, P. A. Davey, N. Kanwal, N. R. Baker, T. Lawson

**Affiliations:** ^1^School of Biological Sciences, University of Essex, Wivenhoe Park, Colchester, Essex CO4 3SQ, UK; ^2^School of Computing and Engineering Science, University of Essex, Wivenhoe Park, Colchester, Essex CO4 3SQ, UK

**Keywords:** Chlorophyll fluorescence imaging, dynamic responses, leaf heterogeneity, screening, thermal imaging, water use efficiency.

## Abstract

Instrumentation and methods for rapid screening and selection of plants with improved water use efficiency are essential to address current issues of global food and fuel security. A new imaging system that combines chlorophyll fluorescence and thermal imaging has been developed to generate images of assimilation rate (*A*), stomatal conductance (*g*_s_), and intrinsic water use efficiency (WUE_i_) from whole plants or leaves under controlled environmental conditions. This is the first demonstration of the production of images of WUE_i_ and the first to determine images of *g*_s_ from themography at the whole-plant scale. Data are presented illustrating the use of this system for rapidly and non-destructively screening plants for alterations in WUE_i_ by comparing *Arabidopsis thaliana* mutants (OST1-1) that have altered WUE_i_ driven by open stomata, with wild-type plants. This novel instrument not only provides the potential to monitor multiple plants simultaneously, but enables intra- and interspecies variation to be taken into account both spatially and temporally. The ability to measure *A*, *g*_s_, and WUE_i_ progressively was developed to facilitate and encourage the development of new dynamic protocols. Images illustrating the instrument’s dynamic capabilities are demonstrated by analysing plant responses to changing photosynthetic photon flux density (PPFD). Applications of this system will augment the research community’s need for novel screening methods to identify rapidly novel lines, cultivars, or species with improved *A* and WUE_i_ in order to meet the current demands on modern agriculture and food production.

## Introduction

One of the greatest challenges plant scientists currently face is global food and fuel security. Water availability is a major constraint of crop yield (Sinclair and Rufty, 2012) and is the single most important factor limiting food production, with significant yield losses reported under water deficit (Boyer, 1982; Mueller *et al.*, 2012; van Ittersum *et al.*, 2012). In order to combat the predicted impacts of increasing drought episodes on crop yield, there is an urgency to identify plants and the underlying mechanisms for improved water use efficiency (WUE). Unfortunately, a major constraint for such crop improvements is the ability to monitor WUE rapidly and non-destructively. It is essential that new techniques and approaches are developed for phenotyping (Fiorani and Schurr, 2013) and to screen for limitations in WUE.

Traditional methods of determining WUE that quantify plant yield or biomass relative to the amount of water used (Stanhill, 1986; Bacon, 2004; Passioura, 2004) are unsuitable for rapid screening for several reasons. These agronomic techniques are not only destructive but also rely on an integrated measurement of biomass/yield at the end of the growing season relative to the amount of water used over the growth period (Chaerle *et al.*, 2005; Morison *et al.*, 2008). Carbon isotope discrimination (Farquhar *et al.*, 1982) has been successfully used to identify crop cultivars with greater WUE (Condon *et al.*, 2004); however, this technique also relies on an integrated measure of WUE over a period of plant growth. Additionally, the technique does not provide an indication of whether differences in WUE are driven by CO_2_ assimilation (*A*) or water loss (Farquhar *et al.*, 1989; Jones, 2004*b*), although the incorporation of oxygen isotope measurements can provide an indication of rates of evaporation from the leaf surface (Farquhar *et al.*, 1998; Barbour, 2007). Leaf-level gas exchange measurements of the rate of *A* relative to transpiration provide an immediate and non-destructive measure of instantaneous WUE (Penman and Schofield, 1951) or ‘intrinsic water use efficiency’ (WUE_i_), when stomatal conductance (*g*_s_) is used instead of transpiration as a measure of water loss (Meidner and Mansfield, 1968; Jones, 1998). Although this approach is flexible in term of the time scale of when measurements can be made, an infrared gas analyser (IRGA) can only take singular measurements on one plant or leaf, at one point in time. Thus, all of the techniques described above to assess WUE have limitations as screening tools, as they tend to be time-consuming and/or destructive. Additionally, biomass and carbon isotope measurements only provide a lifetime measure of WUE based on cumulative seasonal conditions, which may mask specific phenotypic traits that could be advantageous in future breeding programmes (Weyers and Meidner, 1990; Chaerle *et al.*, 2005). Using a combined chlorophyll fluorescence and thermography imaging approach, a non-invasive, high-throughput, high resolution tool has been developed to screen WUE_i_ based on calculated images of *A* and *g*_s_ produced from measurements of photosynthetic efficiency (*F*_q_′/*F*_m_′) and leaf temperature, respectively.

Chlorophyll fluorescence has long been used to examine various photosynthetic parameters in leaves (Baker, 2008) and it is well established that the operating efficiency of photosystem II (PSII; *F*_q_′/*F*_m_′) is related to changes in CO_2_ assimilation in leaves (Genty *et al.*, 1989, 1990; Krall and Edwards, 1990; Cornic and Ghashghaie 1991; Edwards and Baker, 1993; Siebke *et al.*, 1997). However, the relationship is complex and depends on the surrounding gaseous environmental conditions. In C_3_ plants, the relationship between the operating efficiency of PSII is linearly related to photosynthetic CO_2_ fixation of leaves, but only when photorespiration is inhibited and CO_2_ assimilation represents the major sink for the end-products of electron transport, namely ATP and NADPH (Baker and Oxborough, 2004; Baker, 2008). Chlorophyll fluorescence images taken on C_3_ plants under a low [O_2_] (20 mmol mol^–1^) can be converted to images of CO_2_ assimilation using suitable calibrations (Di Marco *et al.*, 1990; Genty *et al.*, 1990; Cornic and Ghashghaie, 1991; Cornic, 1994; Genty and Meyer, 1995).

Infrared thermography (IRT) provides a powerful imaging tool for rapidly, non-invasively, and remotely measuring leaf temperature as a surrogate for *g*_s_ (Omasa *et al.*, 1981; Hashimoto *et al.*, 1984; Jones, 1999). Leaf temperature depends on evaporative cooling, and is a function of *g*_s_ (Jones, 1992). For example, leaf temperature increases as stomata close and restrict evaporative water loss. Thermography has been used for rapid screening of genotypic variation in *g*_s_ (Wang *et al.*, 2004) as well as early diagnosis of drought stress-induced changes in *g*_s_ (Grant *et al.*, 2006; Morison *et al.*, 2008). In the last 20 years there has been increasing interest in quantitative evaluation of stomatal conductance from measurements of leaf temperature, using the basic energy balance equations (Jones, 1999, 2004*a*; Leinonen *et al.*, 2006; Grant *et al.*, 2007). The use of thermography to determine *g*_s_ has been optimized through the development of standard protocols which take into account the surrounding environment, and even the distribution of stomata between the two leaf surfaces (Jones, 1999; Guilioni *et al.*, 2008). Thermography has become a standard technique to determine *g*_s_ in both glasshouse (Grant *et al.*, 2006) and field environments (Grant *et al.*, 2007).

Here the development of a novel imaging system that incorporates measurements of chlorophyll fluorescence and thermal imaging under controlled gaseous conditions is described. Chlorophyll fluorescence images of *F*_q_′/*F*_m_′ provide a quantitative image of *A*, whilst images of leaf temperature are converted to *g*_s_ using well-defined methods that take into account the surrounding environment. Further manipulation of these two images provides for the first time an image of intrinsic water use efficiency (*I*WUE_i_=*A*/*g*_s_). Previous researchers have used combined chlorophyll fluorescence and thermal imaging approaches to evaluate photosynthetic performance in relation to stomatal behaviour (e.g. Chaerle *et al.*, 2005, 2007; Lawson, 2009; Glenn, 2012), but the majority of these studies have been carried out at the leaf or tissue scale (Omasa and Takayama, 2003; Messinger *et al.*, 2006) and have not been used to determine WUE, but have been focused on physiological analysis of mechanisms that coordinate responses observed between mesophyll photosynthesis and stomatal behaviour. A major advantage of the combined imaging approach reported here is the ability for multiple samples to be measured at any one time and the fact that spatial heterogeneity within plants and leaves can be readily identified. Differences between wild-type (WT) and open stomatal mutant (OST1-1; Merlot *et al.*, 2002) *Arabidopsis thaliana* plants are demonstrated using the system, with spatial and temporal heterogeneity in *A*, *g*_*s*_, and *I*WUE_i_ being observed in the images. It is important to take into account such heterogeneity as it is well established that *g*_s_ and photosynthesis are not uniform over a leaf surface (Oxborough and Baker, 1997; Weyers and Lawson, 1997; Weyers *et al.*, 1997; Mott and Buckley, 1998; Lawson *et al.*, 2002; Peak *et al.*, 2004; West *et al.*, 2005; Kamakura *et al.*, 2012) and that such heterogeneity is also dynamic (Lawson and Weyers, 1999) often being driven by variations in the microenvironment (Flexas and Medrano, 2002; Peak *et al.*, 2004; Lawson *et al.*, 2012). The system has not only been constructed to image spatial differences in WUE_i_, but it has been specifically designed to facilitate dynamic measurements, which allow the impact of changing environmental conditions on stomatal behaviour to be assessed in relation to photosynthetic performance and WUE.

## Materials and methods

### Plant material

*Arabidopsis thaliana* genotypes Columbia-0 (Col-0), Wassilewskija-0 (Ws-0), and Landsberg erecta (Ler), and the mutant *Open Stomata 1* (OST1*-*1) were grown in a controlled environment at 23 °C and 1.1 kPa vapour pressure deficit (VPD) day and night. The photoperiod was 8/16h light/dark with a photosynthetically active photon flux density (PPFD) of 135±10 µmol m^–1^ s^–1^. Two-week-old seedlings were transferred either to 100cm^3^ pots or to 6-well culture plates (Nunc, Roskilde, Denmark) containing compost (Levington’s F2S, Everris, Ipswich, UK). Plants were maintained under well-watered conditions. A layer of vermiculite (Vermiculite Lite, Sincair, UK) was placed over the compost surface of plants grown in well plates to improve the contrast between the plants and the background during imaging.

*Phaseolus vulgaris* L. cv. ‘Evergreen’ were grown in a temperature-controlled glasshouse at 23±4 °C. Lighting was supplemented by sodium vapour lamps (600W; Hortilux Schrèder, The Netherlands) when external solar radiation fell below 500 µmol m^–2^ s^–1^ PPFD, during a 10h period. Plants were grown from seed in 650cm^3^ pots containing compost (Levington’s F2S) and watered every 2 d with Hoagland’s nutrient solution.

### Combined chlorophyll fluorescence and thermal imaging system

**Fig. 1.**
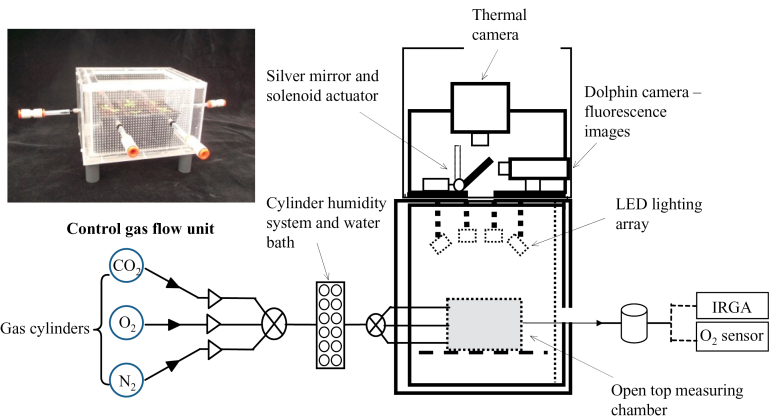
Schematic diagram of the system used to image whole plants for chlorophyll *a* fluorescence and temperature under controlled conditions. The imaging system was modified to allow the attachment of two cameras, the thermal camera being directly positioned above the plant and the fluorescence camera situated at 90 ° to the thermal camera, utilizing a silver-backed mirror at 45 ° to capture images of *F*_q_′/*F*_m_′. The plant was positioned within an open-topped chamber (photograph insert) where concentrations of O_2_, CO_2_, and H_2_O were maintained at a typical flow rate of ~0.87 l s^–1^ N_2_, with all gases passing through a humidifying system prior to entering the chamber. Concentrations of O_2_, CO_2_, and H_2_O were measured every second by an IRGA and oxygen sensor.

A chlorophyll *a* fluorescence imaging system (FluorImager, Technologica, Colchester, Essex, UK), previously described by Barbagallo *et al.* (2003), was modified by repositioning the camera from being directly above the chamber to a 90 ° angle, whilst maintaining the same distance from the subject material (Fig. 1). A silver-coated mirror (Thor-Optics, Dachau, Germany) was hinged on an axis directly above the original camera port. At a 45 ° angle, the mirror reflected the chlorophyll *a* fluorescence signal directly onto the camera. For thermal imaging, a thermal camera (TH7100 Thermal Tracer, NEC Avio Infra-red Technologies Co. Ltd, Japan) was positioned in the original location of the chlorophyll fluorescence camera, directly above the imaging port. Pivoting the mirror allowed thermal and chlorophyll fluorescence images to be captured within 2 s of each other (Fig. 1). The thermal camera has a temperature resolution of 0.1 °C, and all measurements were made at a distance of 0.45 m and emissivity (ε) of 0.98.

### Gas control in imaging chamber

In order to control concentrations of O_2_, CO_2_, and H_2_O vapour during imaging, an in-house designed chamber was constructed. As the spectral wavelength used for chlorophyll fluorescence excitation and thermal and fluorescence emission span from the visible to the infrared (450–14 000nm), a chamber window could not be used and instead an open-top design was employed. The chamber was built from Perspex with inner dimensions of 145mm (length)×105mm (width)×95mm (depth), including a 10mm width flange on the top surface. With the exception of the base, the chamber consisted of an inner and outer wall separated by a 10mm gap. The outer walls were connected on each of the four sides by 6mm PTFE tubing connections. The inner wall was perforated with 1mm diameter holes at a density of 9 per 100mm^2^, which was optimal for maintaining homogenous gas concentrations whilst minimizing leaf movement through turbulence. Total gas flow entering the chamber was typically 0.87 l s^–1^. Within the chamber, target gas concentrations of N_2_, O_2_, and CO_2_ were individually maintained by mass flow controllers (EL Flow, Bronkhorst, Ruurlo, The Netherlands), connected to compressed gas cylinders containing 100% N_2_, O_2_, and CO_2_, respectively (British Oxygen Company-Industrial Gases, Ipswich, UK). In order to control water vapour concentration, gas was bubbled through temperature-regulated gas wash bottles (Cole-Parmer, London, UK) prior to entering the chamber.

Gas composition in the chamber was monitored at plant height by sampling air with a diaphragm pump (Type 124, ADC Hoddesdon, Herts, UK) at 500cm^3^ min^–1^. Oxygen concentration was measured with a flow-through oxygen sensor (S101, Qubit Systems, Kingston, Canada) which was calibrated using O_2_-free air and a 205 mmol mol^–1^ [O_2_] standard (British Oxygen Company-Industrial Gases). Both CO_2_ and H_2_O vapour concentrations were measured with an IRGA (Li- 840, Li-Cor, NE, USA) calibrated weekly using a standard gas for CO_2_ (±2.5% tolerance) (British Oxygen Company) and a dewpoint generator (LI-610; Li-Cor) for H_2_O vapour.

**Fig. 2.**
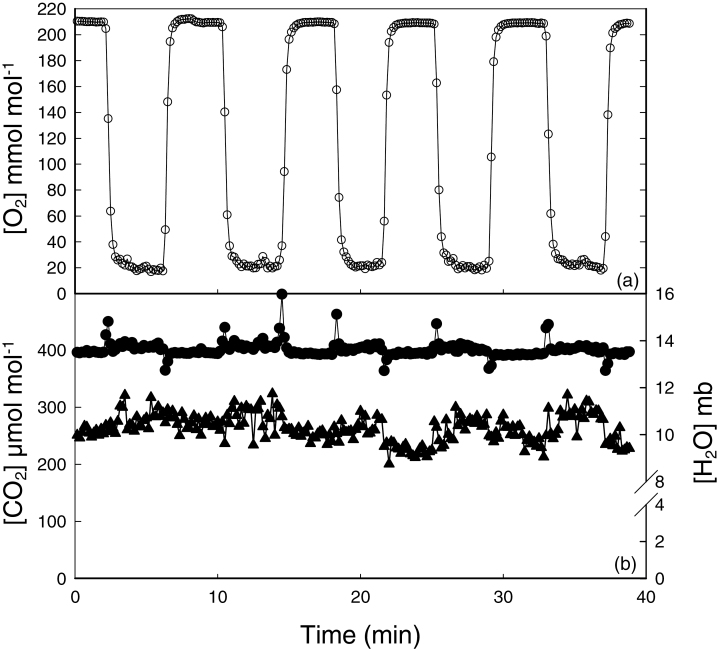
Concentrations of (a) O_2_ and (b) CO_2_ (filled circles) and water vapour (triangles) in the measuring chamber during an experiment. Oxygen concentration was switched from atmospheric (210 mmol mol^–1^) to 20 mmol mol^–1^ five times while maintaining CO_2_ and H_2_O vapour concentrations.

Since *F*_q_′/*F*_m_′ is only linearly related to *A* when photorespiration is inhibited, it was essential that [O_2_] within the chamber could be reduced and maintained at 20 mmol mol^–1^ during the imaging process and immediately returned to ambient concentration when complete. Figure 2a shows that [O_2_] could be rapidly reduced from 205 mmol mol^–1^ to 20 mmol mol^–1^ within <50 s of switching the gas input. In this example, low [O_2_] were maintained (within 2 mmol mol^–1^ of the target value) for several minutes, although typically <20 s were required to capture an image of *F*_q_′/*F*_m_′. Once an image was taken, the [O_2_] was returned to ambient within 30 s (Fig. 2a). Figure 2b illustrates the stability of [CO_2_] (400 µmol m^–2^ s^–1^ ±5%) and H_2_O concentrations (±10% of target values) during the [O_2_] changes and for the duration of the experimental procedure.

### Estimating carbon assimilation from chlorophyll fluorescence parameters

*F*_q_′/*F*_m_′ is calculated from measurements of steady-state fluorescence in the light (*F*′) and maximum fluorescence in the light (*F*_m_′) since *F*_q_′/*F*_m_′=(*F*_m_′–*F*′)/*F*_m_′. Images of *F*′ were taken when fluorescence was stable at the desired PPFD, whilst images of maximum fluorescence were obtained after a saturating 800ms pulse of 5500 µmol m^–2^ s^–1^ PPFD (Oxborough and Baker, 1997, 2000; Baker *et al.*, 2001). The intensity of the saturating pulse is sufficient to saturate PSII in relatively low PPFD-grown plants, but may need to be increased or modified for high PPFD-grown samples (see Loriaux *et al.*, 2013). Using the first fully expanded leaves of 5-week-old *A. thaliana* (Ws-0), *F*_q_′/*F*_m_′ and *A* were measured at 15 different CO_2_ concentrations. Leaf intercellular [CO_2_] (*C*_i_) and *A* were measured using an IRGA (CIRAS-1, PP Systems, Amesbury, USA) and *F*_q_′/*F*_m_′ was determined from simultaneous images of chlorophyll fluorescence. These data were used to produce *A*/*C*_i_ and *F*_q_′/*F*_m_′/*C*_i_ response curves, that were used to calibrate *F*_q_′/*F*_m_′ with net CO_2_ assimilation (Morison *et al.*, 2005). The standard cuvette window of the IRGA was replaced with non-reflective glass to enable fluorescence images to be taken of leaves. At each external CO_2_ concentration (*C*_*a*_), *C*_i_ and *A* were allowed to reach steady state before images were captured. Measurements started at ambient *C*_*a*_ of 400 µmol mol^–1^, before *C*_*a*_ was decreased step-wise to a lowest concentration of 50 µmol mol^–1^, then increased step-wise to an upper concentration of 2000 µmol mol^–1^. Leaf temperature and VPD were maintained at 25 °C and 1.2 kPa, respectively. Four replicate *A*/*C*_i_ and *F*_q_′/*F*_m_′/*C*_i_ response curves were measured at 200, 500, and 800 µmol m^–2^ s^–1^ PPFD. Plots of *F*_q_′/*F*_m_′ against *A* over the *C*_i_ range and at these different PPFDs were used to determine the relationships between measured *A* and *F*_q_′/*F*_m_′.

### Estimating stomatal conductance from leaf temperature

IRT has been widely recognized to be a powerful imaging tool, capable of rapidly and non-invasively measuring leaf temperatures (Jones, 1999, 2004*a*; Leinonen *et al.*, 2006; Grant *et al.*, 2007). As leaf temperature is dependent on evaporative cooling, it can be used as an indirect measure of leaf conductance to water vapour (*g*_l_) or its reciprocal, leaf resistance (*r*_w_) (Jones, 1992, 1999; Guilioni *et al.*, 2008). Although this measure of leaf conductance also includes the water lost through the cuticle, termed cuticle conductance (*g*_c_), the value is relatively small and usually neglected, subsequently the terms leaf and stomatal conductance (*g*_s_) are used interchangeably (see Jones, 1999). Leaf temperature also depends on the environmental conditions around the leaf (Jones, 1992, 2004*a*) and, in order to determine *g*_s_ from thermal images, an estimation of the boundary layer resistance to water vapour (*r*_aw_) is needed along with known wet and dry temperature reference standards (Jones, 1999). Two temperature references are required, one that provides an infinite resistance to water vapour (e.g. leaf material greased on both sides), whilst the second provides a near-zero resistance to water vapour (e.g. leaf surface painted with a detergent–water mix). The temperature standards normalize the measured leaf temperature to the environmental conditions surrounding it and it is assumed that these surfaces have the same radiative properties (Jones, 2004*a*). The application of the wet standard to either one or both sides of the leaf is used to account for the distribution of the stomata (Guilioni *et al.*, 2008). In this study, a one-sided wet standard was used for *Phaseolus vulgaris* leaves and a two-sided wet standard was used for *A. thaliana* leaves.

Leaf resistance to water vapour (*r*_w_) was calculated from thermal images (Guilioni *et al.*, 2008) for anisolateral leaves using the following equation:


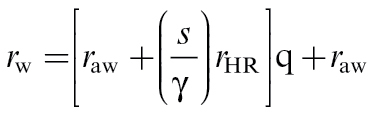


where *r*_aw_ is the leaf boundary layer resistance to water vapour, *s* is the slope of the curve relating saturation vapour pressure to temperature, and γ is the psychrometric constant. *r*_HR_ represents the parallel resistance to heat and radiative transfer, and θ is the temperature phrase, normalizing leaf temperature with the wet and dry standards; 
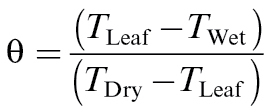
,where *T*_Leaf_, *T*_Wet_, and *T*_Dry_ are the mean temperatures of the leaf wet and dry standards, respectively. This temperature phrase normalizes leaf temperature (*T*_Leaf_) between a surface of lower resistance and temperature (*T*_Wet_) and a surface of higher temperature and a resistance greater than that of the leaf (*T*_Dry_). Estimates of *g*_s_ were made from the reciprocals of *r*_w_; *g*_s_=1/ *r*_w_.

In order to test how robust the method was for estimating *g*_s_ from leaf temperature, IRGA measurements of *g*_s_ were taken alongside estimates determined from images of leaf temperature from *P. vulgaris* and *A. thaliana* (WS-0 and Col-0). *r*_aw_ values were calculated using damp filter paper leaf replicates with areas of 0.0025 m^2^ and 0.00044 m^2^ for *P. vulgaris* and *A. thaliana*, respectively, following the vapour-flux density method of Weyers and Meidner (1990).

The rate of water loss from the leaf replicates was determined under the imaging system from the change in weight (±1mg), measured every 30 s for a period of 15min. The average surface temperature of the filter paper was analysed using thermal imager software (Radiometric Thermography Studio Complete, Metrum, Wokingham, UK) to monitor temperature stability throughout the measurement period. VPD was maintained at 1.7 kPa (±0.006 SE) and air temperature at 21.7 °C (±0.04 °C SE). Concurrent measurements of air speed (average 0.11 m s^–1^) over the filter paper were made using a hot-wire anemometer (Model 425, Testo, Alton, Hampshire, UK).

Leaves of *P. vulgaris* were kept flat by a wire support, with the adaxial surface facing upwards. Five-week-old *A. thaliana* plants were maintained in pots large enough to ensure leaves were parallel to the soil surface. A 30% Tween (Sigma, Gillingham, Kent, UK) solution or vacuum grease (Dow Corning, Midland, Michigan, USA) were applied to different 1cm^2^ areas of the leaf to create the wet and dry standards, respectively (see above). To generate a range of stomatal conductances, plants were left for 30min to reach steady state at PPFDs ranging from 100 µmol m^–2^ s^–1^ to 1000 µmol m^–2^ s^–1^. The plant was then placed under the imaging system and a thermal image was taken followed immediately by an IRGA measurement. Conditions inside the imager were maintained at 1.7 kPa VPD and 21.7 °C air temperature.

### Construction of images of water use efficiency

**Fig. 3.**
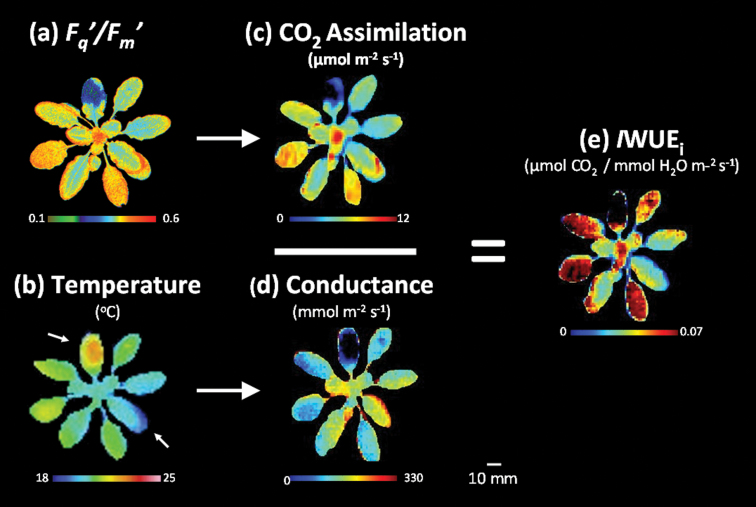
Typical (a) *F*_q_′/*F*_m_′ and (b) temperature images of an *A. thaliana* plant illustrating the production of (c) CO_2_ assimilation (*A*), (d) stomatal conductance (*g*_s_), and (e) intrinsic WUE (*I*WUE_i_) images. Calibrations were applied to convert pixel values within each image into *A* and *g*_s_ data, respectively. The wet and dry temperature standards used in the calculation of *g*_s_ are indicated by arrows on the temperature image. To produce an image of WUE_i_ (*A*/*g*_s_=*I*WUE_i_), the *A* image was rotated, scaled, and interpolated onto the image of *g*_s._ The colour bar beneath each image shows the range of parameter values. For further details of this process, see Materials and methods.

The rationale and approach to constructing images of WUE_i_ (*I*WUE_i_) is illustrated in Fig. 3. Image values of *F*_q_′/*F*_m_′ (Fig. 3a) and leaf temperature (Fig. 3b) are converted to *A* (Fig. 3c) and *g*_s_ (Fig. 3d) using the calibrations described above. To produce the image of *I*WUE_i_, the *A* image (Fig. 3c) was rotated, scaled, and interpolated to re-map spatially pixel values of *A* to values of *g*_s_. At the individual pixel level, values of *A* were divided by values of *g*_s_ to produce pixel values of *I*WUE_i_ (Fig. 2e; *A*/*g*_s_=*I*WUE_i_).

### Software development for image construction

**Fig. 4.**
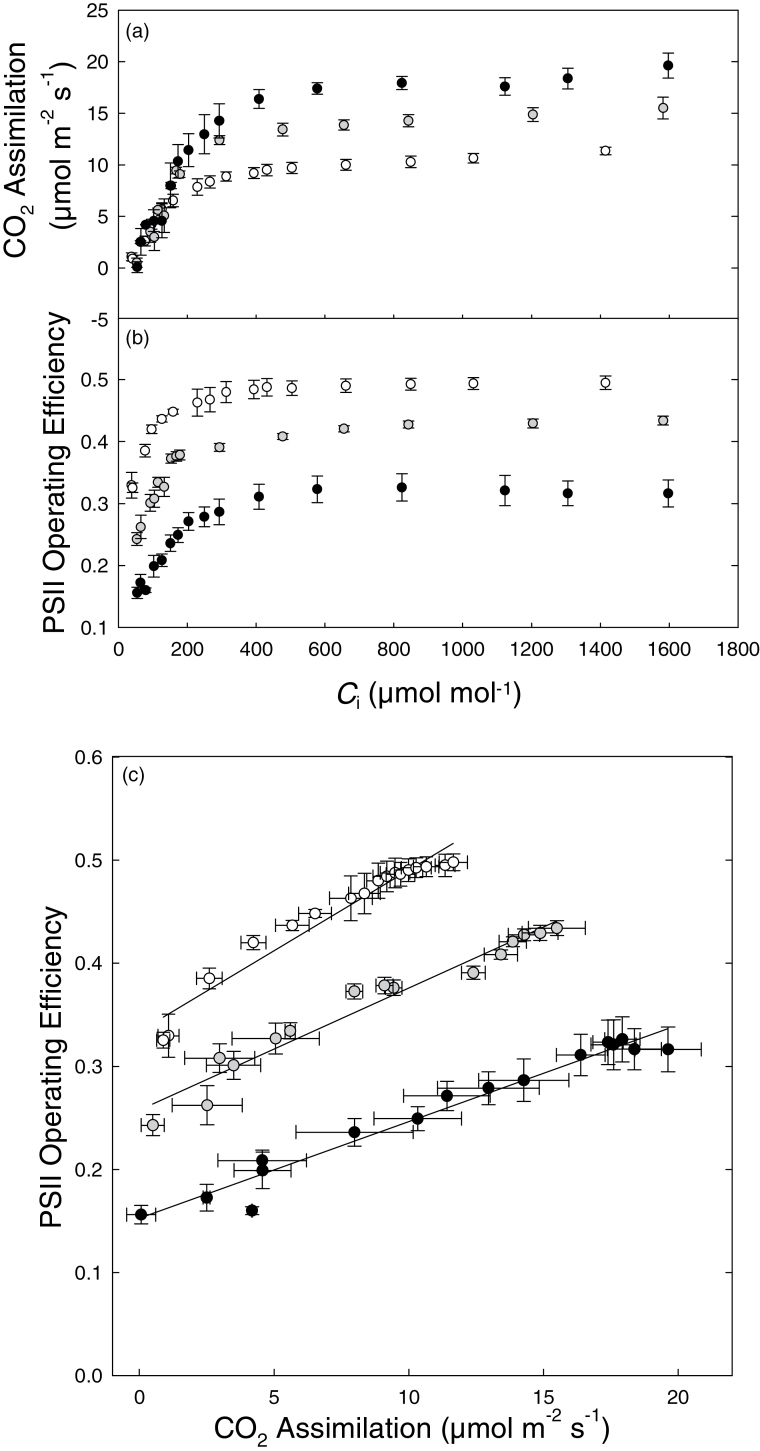
The response of CO_2_ assimilation (a) and PSII operating efficiency (b), estimated from *F*_q_′/*F*_m_′, to changes in internal CO_2_ concentration (*C*_i_) under 20 mmol mol^–1^ O_2_ and at 200 (open circles), 500 (grey circles), and 800 (filled circles) µmol m^–2^ s^–1^ PPFD. The correlation between CO_2_ assimilation and PSII operating efficiency (c) was fitted using a linear regression for each PPFD intensity. All measurements were made on 5-week-old *A. thaliana.* Air temperature and VPD were 25 °C and 1.2 kPa, respectively. Data are the means with standard errors (*n*=3–5).

In-house specifically designed software (ImFluTem; IFT) with a graphical user interface (GUI) was developed using Matlab (Mathworks, Natick, MA, USA) to facilitate data processing and construction of images of WUE_i_. Leaf temperature and *F*_q_′/*F*_m_′ data were downloaded from their respective commercial image capture programs and stored in a *x*,*y*,*z* matrix format which were uploaded into the Matlab program as text files. The maximum image area for leaf temperature images was 150cm^2^ (240×320 pixels) whilst chlorophyll fluorescence images were smaller (90cm^2^) with a greater pixel resolution (700×520 pixels). The program allowed users to select the PPFD used and to map the data to *A* using the appropriate calibration (see Fig. 4). It also allowed manual input of other environmental variables (including air temperature, wet and dry reference temperature, and boundary layer resistance) required for the conversion of leaf temperature data into *g*_s_. Alternatively, an option was available that allowed user selection of areas of the images that corresponded to wet and dry standard reference material. As mentioned above, *A* images were rotated, scaled, and interpolated within the program to align pixels between the two images. Although the majority of the scaling and rotation values were embedded in the coding of the program, manual input options were designed to allow users full control over the mapping of images. Frequency distribution of the raw data provided a reference check for the range of interpolated and calculated values of the new images produced, with the removal of any pixel values falling out of the original distribution. Images of *I*WUE_i_ were constructed from individual pixels values of *A* divided by their corresponding pixel value of calculated *g*_s_ to produce pixel values of *I*WUE_i_. Values were mapped to a colour pallet that corresponded to 10 evenly distributed data bins determined by user-defined maximum and minimum values. Output data were stored as text files along with corresponding JPEG images of *A*, *g*_s_, and *I*WUE_i_. An additional GUI assisted with processing multiple images (particularly imported when dynamic protocols were employed) and area selection for analysis. Multiple files were uploaded using a wildcard input function. The images and data generated used the same numbered wild card function as the original file.

### Screening for differences in water use efficiency

Three (4-week-old) plants of *A. thaliana* WT (Ler) and three OST1-1 mutants grown in a well plate were dark adapted for 20min before being placed in the imaging system and exposed to a PPFD of 200 µmol m^–2^ s^–1^. After a minimum of 20min and at stable *F*′, the [O_2_] was decreased from 205 mmol mol^–1^ to 20 mmol mol^–1^ for ~50 s and a thermal image recorded. This was immediately followed by the application of a saturating pulse to produce *F*_m_′ allowing calculation of *F*_q_′/*F*_m_′. Following these measurements, [O_2_] was returned to 205 mmol mol^–1^. A second set of measurements was taken after a further 15min. All images were made at a [CO_2_] of 400 µmol mol^–1^, VPD of 1.1 kPa, and air temperature of 21 °C. A 1cm^2^ greased circle of leaf material provided the dry standard (*T*_Dry_) while damp filter paper, the same shape and size as a fully expanded leaf, provided the wet standard (*T*_Wet_). Estimates of *I*WUE_i_ were made as described above.

### Monitoring of dynamic changes in water use efficiency

To image the dynamic response of *A*, *g*_s_, and *I*WUE_i_ to a step-wise increase in light, 5-week-old *A. thaliana* plants were positioned in the imaging chamber at 200 µmol m^–2^ s^–1^ PPFD, 400 µmol mol^–1^ [CO_2_], 1.1 kPa VPD, and 21 °C air temperature. A 1cm^2^ patch of grease was applied to the abaxial and adaxial surface of a leaf to provide the dry standard, whilst damp filter paper was used for the wet standard. Every 3min, [O_2_] was reduced from 205 mmol mol^–1^ to 20.5 mmol mol^–1^ and thermal and fluorescence images taken. After 15min, PPFD was increased to 800 µmol m^–2^ s^–1^ and images recorded every 3min for a further 30min. To compare *A*, *g*_s_, and *I*WUE_i_ values calculated from the images with those obtained using standard IRGA, measurements were made concurrently on similar plants of the same age grown and treated under the same conditions.

### Comparison of water use efficiency determined by imaging and gas exchange

IRGA measures of WUE_i_ were made on 5-week-old *A. thaliana* plants at 200 µmol m^–2^ s^–1^ or 800 µmol m^–2^ s^–1^ PPFD and a range of *C*_i_ values (35–1400 µmol mol^–1^). Air temperature and VPD were maintained at 25 °C and 1.2 kPa, respectively. Immediately after an IRGA reading was taken, the plant was rapidly transferred to the imaging system that was maintained at the same environmental conditions as the leaves in the IRGA chamber, with the exception of [O_2_] that was maintained at 20.5 mmol mol^–1^. When *F*′ was stable, thermal and fluorescence images were taken and used to calculate *I*WUE_i_.

## Results

### Relationship between F_q_′/F_m_′ and CO_2_ assimilation for leaves in imaging chamber

The response of *F*_q_′/*F*_m_′ and *A* to changing internal [CO_2_] (*C*_i_) at 20 mmol mol^–1^ [O_2_] and at the different PPFDs is shown in Fig. 4a and b. As photorespiration was suppressed, both *A* and *F*_q_′/*F*_m_′ showed similar shaped saturation functions of *C*_i_, with an initial linear increase as *C*_i_ increases before they plateau at a given *C*_i_, which is dependent on PPFD. A plot of *A* against *F*_q_′/*F*_m_′ (Fig. 4c) shows a robust linear relationship between the two parameters at each of the three PPFDs. Regression analysis between the two parameters provided the functional relationship with which measurements of *F*_q_′/*F*_m_′ were converted to *A*, and the regression coefficients indicated that >94% of the variation was accounted for in the relationships.

### Validity of estimating stomatal conductance from thermal images

**Fig. 5.**
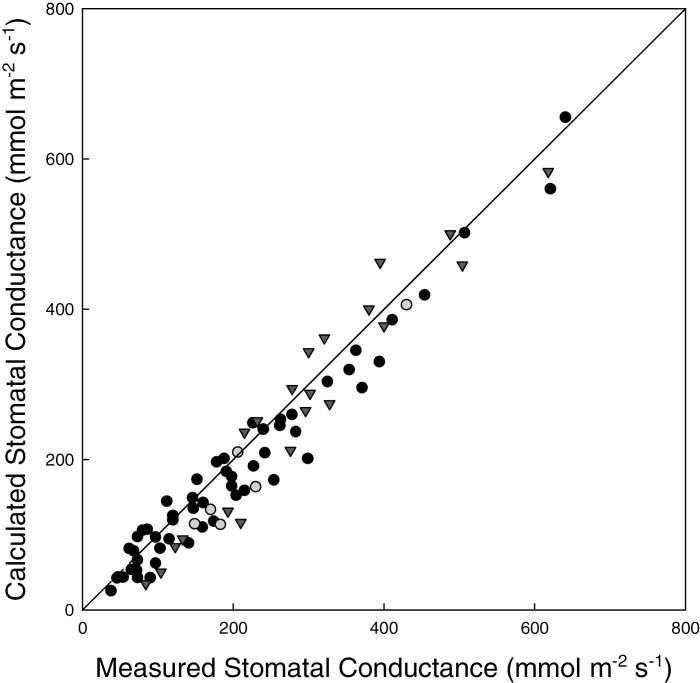
A comparison between stomatal conductance calculated from thermal images with that measured using an IRGA. To stimulate a range of conductances, leaves of *Phaseolus vulgaris* (filled circles) and *A. thaliana thaliana* Col-0 (grey circles) and WS-0 (triangles) were exposed to PPFDs between 200 µmol m^–2^ s^–1^ and 2000 µmol m^–2^ s^–1^. The solid line represents a 1:1 relationship (*P* < 0.0001, *R*_s_=0.96).

Stomatal conductance calculated from images of leaf temperature were compared with independent measurements of *g*_*s*_ obtained by infrared gas exchange analysis (Fig. 5). These data demonstrated a significant correlation between measured and calculated *g*_*s*_ ranging from 40–640 mmol m^–2^ s^–1^ to 640 mmol m^–2^ s^–1^, for both plant species. This relationship validates the use of thermography to evaluate *g*_s_ accurately.

### Demonstration of use of fluorescence and thermal imaging to detect differences in water use efficiency

**Fig. 6.**
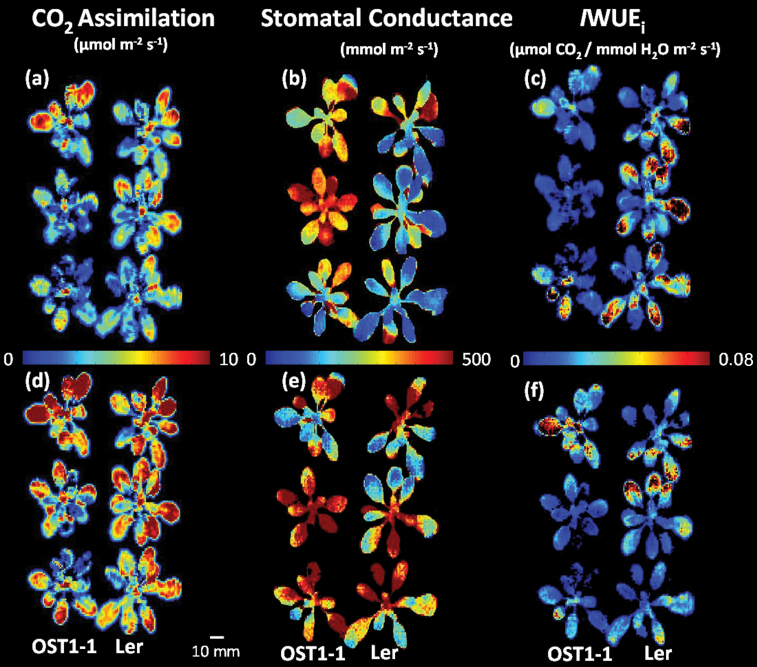
Screening of mutant OST1-1 (left column of three plants) and wild-type Ler (right column of three plants) for differences in CO_2_ assimilation (*A*), stomatal conductance (*g*_s_), and *I*WUE_i_. The plants were dark adapted for 20min before the PPFD was increased to 200 µmol m^–2^ s^–1^. Images were taken after 20min (a–c) and after 35min (d–f). Images of *A* (a and d), *g*_*s*_ (b and e), and *I*WUE_i_ (c and f) were calculated. All images were captured at 400 µmol mol^–1^ [CO_2_], 21 °C air temperature, and 1.1 kPa VPD. The colour bar between each image shows the range of parameter values.

Images of *A* (Fig. 6a, d) and *g*_s_ (Fig. 6b, e) determined from images of *F*_q_′/*F*_m_′ and leaf temperature, respectively, were used to calculate images of *I*WUE_i_ (Fig. 6c, f) for WT and OST1-1 *A. thaliana* measured after 20min and 35min of applying 200 µmol m^–2^ s^–1^ PPFD. After 20min (*T*_20_) in the light, there was no significant difference in *A* between the WT and OST1-1 (Fig. 6a), with all plants showing a mean value of 3.1 µmol m^–2^ s^–1^. OST1-1 exhibited a lower *A* than the WT, although the difference was not significant. The distribution of pixel values within each image indicated that the majority of OST1-1 values were below 4 µmol m^–2^ s^–1^ (Supplementary Fig. S1 available at *JXB* online), whereas WT values covered a greater distribution range (0.5–8 µmol m^–2^ s^–1^) and were more evenly distributed within it. Stomatal conductance was significantly (*P*=0.069) higher in OST1-1 plants (Fig. 6b); mean *g*_s_ values for OST1-1 were 395 mmol m^–2^ s^–1^ which were 31% greater than the average WT values. This difference was also reflected in the distribution of values within the images (see Supplementary Fig. S1). The mutant showed a frequency distribution that was skewed toward higher conductance values (> 500 mmol m^–2^ s^–1^) compared with the WT, where the greatest frequency of pixel values were <200 mmol m^–2^ s^–1^. The higher *g*_s_ value observed in the OST1-1 plants, in conjunction with no difference in *A*, resulted in a significantly (*P*=0.019) lower *I*WUE_i_ compared with the WT. The average *I*WUE_i_ in WT plants was 44% greater than in the mutants (Fig. 6c). After a further 15min (*T*_35_) at 200 µmol m^–2^ s^–1^ PPFD, *A* in both plant types increased by 1.9 to 5.2 µmol m^–2^ s^–1^ and 4.8 µmol m^–2^ s^–1^ for WT and OST1-1 plants, respectively. Average *g*_s_ in the WT had increased by 43% (118 mmol m^–2^ s^–1^); however, there was no change in mean OST1-1 *g*_s_ (Fig. 6e). The higher *g*_s_ and *A* after 35min in WT plants (Fig. 6d) resulted in these plants having a similar *I*WUE_i_ to the OST1-1 mutants (Fig. 6f). The frequency distribution of pixels reflected the considerable variation observed in the images, with values ranging from 0.5 µmol m^–2^ s^–1^ to 10 µmol m^–2^ s^–1^ for *A*, 500 mmol m^–2^ s^–1^ to 1000 mmol m^–2^ s^–1^ for *g*_s_, and 0.0025 µmol CO_2_/mmol H_2_O m^–2^ s^–1^ to 0.05 µmol CO_2_/mmol H_2_O m^–2^ s^–1^ for *I*WUE_i_ (Supplementary Fig. S1). However no difference in distributions were observed between the two plant types at 35min.

### Imaging dynamic changes in water use efficiency

**Fig. 7.**
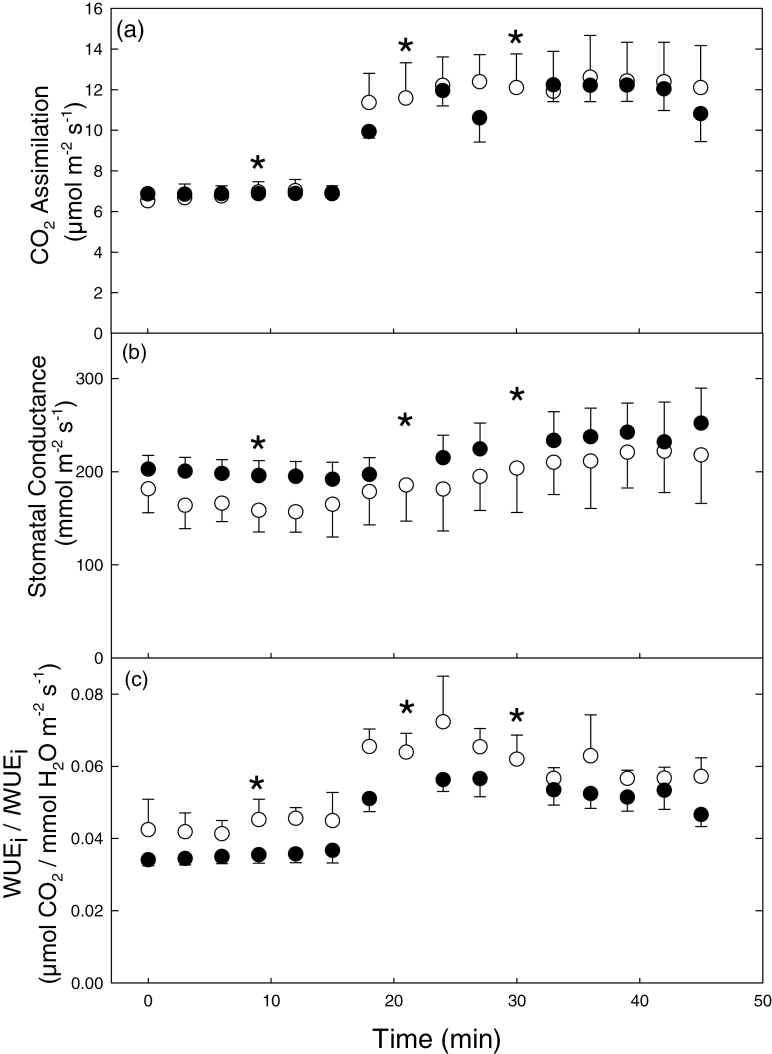
Changes in measured (filled circles) and calculated (open circles) values of CO_2_ assimilation, stomatal conductance, and WUE_i_/*I*WUE_i_ were determined on leaves of 5-week-old *A. thaliana* plants during a stepwise increase in PPFD from 200 µmol m^–2^ s^–1^ to 800 µmol m^–2^ s^–1^ PPFD after 15min (↑). The asterisk (*) denotes data points presented as images in Fig. 9. Air temperature and VPD were 25 °C and 1.2 kPa, respectively. Data are means with standard errors (*n*=3).

**Fig. 8.**
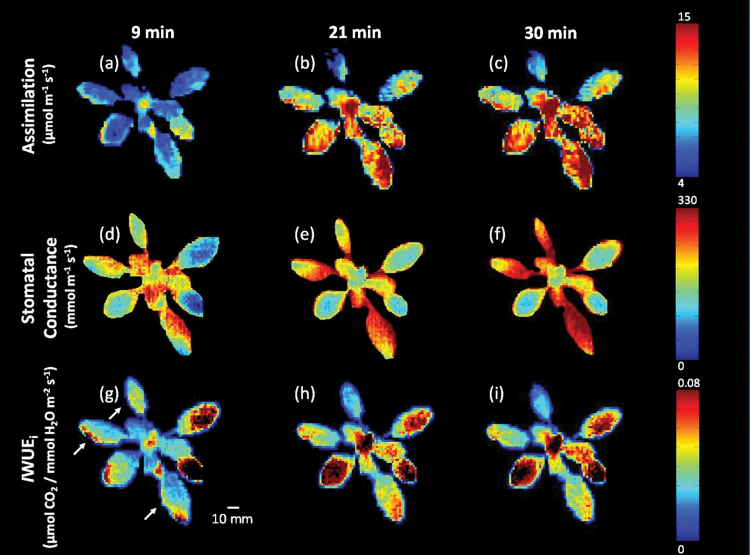
Images of CO_2_ assimilation, stomatal conductance, and *I*WUE_i_ taken at 9, 21, and 30min as shown in Fig. 7. Arrows indicate older leaves selected for analysis. Air temperature and VPD were 25 °C and 1.2 kPa, respectively. The colour bars on the right show the range of parameter values.

**Fig. 9.**
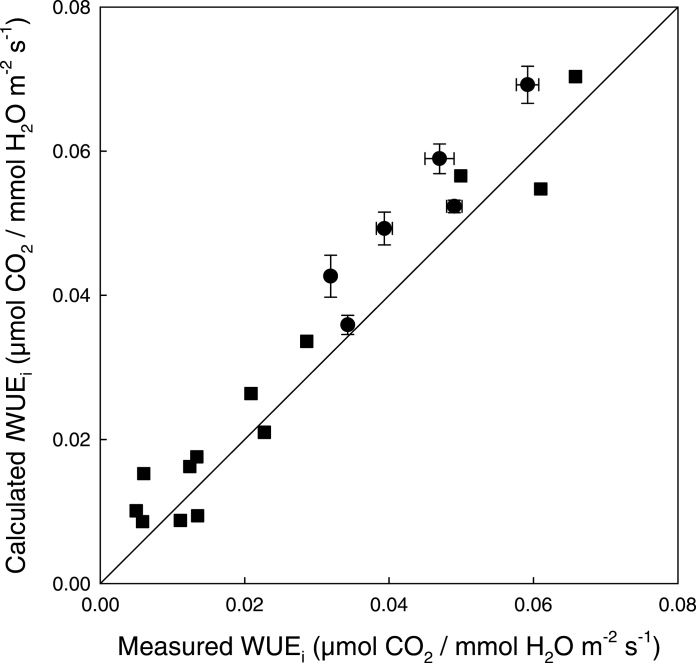
A comparison of IRGA measurements of WUE_i_ and calculated values of *I*WUE_i_ from images captured using the combined imaging system. WUE_i_ was measured from leaves (circles) of *A. thaliana* during a step-wise change in light at a CO_2_ concentration of 400 µmol mol^–1^ (see Fig. 8); data are means with standard errors (*n*=5–8). Measurements were taken from individual leaves (squares) at CO_2_ concentrations between 100 µmol mol^–1^ and 2000 µmol mol^–1^. Air temperature and VPD were 25 °C and 1.2 kPa, respectively. The solid line represents a 1:1 relationship.

WUE_i_ can oscillate rapidly with changes in both *A* and *g*_s_, driven by fluctuations in the environment and in particular light. To demonstrate the effect of changing light on stomatal behaviour and the impacts on *A* and WUE_i_, an *A. thaliana* plant was subjected to a step-wise increase of 600 µmol m^–2^ s^–1^ PPFD following stabilization at 200 µmol m^–2^ s^–1^ PPFD. In order to verify that the values obtained from the images were typical of those obtained using standard gas exchange methods, simultaneous measurements of *A*, *g*_s_, and WUE_i_ were captured using an IRGA (Fig. 7) and the combined imaging system (Fig. 8). There were no significant differences between measurements obtained using the two methods. *A* increased from 6.8 µmol m^–2^ s^–1^ to 11.9 µmol m^–2^ s^–1^ at 800 µmol m^–2^ s^–1^ PPFD (Fig. 7a). At 200 µmol m^–2^ s^–1^ PPFD, steady-state mean *g*_s_ was 165 mmol m^–2^ s^–1^ and 196 mmol m^–2^ s^–1^ in imaged and IRGA measured plants, respectively. Stomatal conductance increased ~30% in both measured and calculated data after 30min at 800 µmol m^–2^ s^–1^ PPFD (Fig. 7b). Although the response of *g*_s_ determined from the imaging system mirrored that of IRGA measurements, the values on average were 13% lower. This discrepancy is most probably due to differences in the size of the boundary layer surrounding the leaves under the imaging system and inside the IRGA cuvette (see Discussion). Steady-state WUE_i_ increased from ~0.036 µmol CO_2_ mmol/H_2_O m^–2^ s^–1^ at 200 µmol m^–2^ s^–1^ PPFD to 0.052 µmol CO_2_/mmol H_2_O m^–2^ s^–1^ at 800 µmol m^–2^ s^–1^ PPFD in measurements obtained using the IRGA (Fig. 7c). *I*WUE_i_ was slightly higher than IRGA measurements at 200 µmol m^–2^ s^–1^ PPFD, with an average value of 0.043 µmol CO_2_/mmol H_2_O m^–2^ s^–1^. *I*WUE_i_ steadily increased after PPFD was increased to 800 µmol m^–2^ s^–1^ PPFD and reached a maximum value of 0.072 µmol CO_2_/mmol H_2_O m^–2^ s^–1^ before stabilizing at a slightly lower value of 0.056 µmol CO_2_/mmol H_2_O m^–2^ s^–1^, identical to the IRGA measurements. *I*WUE_i_ was significantly greater (*P*=0.02) at 800 µmol m^–2^ s^–1^ due primarily to the significant and rapid increase in *A* and the slower, smaller increase in *g*_s_ following the step-wise increase in PPFD (Fig. 7c). The small non-significant differences observed between measured and calculated values were most probably due to the heterogeneity observed between leaves and across plants. This variation cannot be taken into account by IRGA measurements of individual leaves. Images taken at 9, 21, and 30min illustrate considerable variation in *A*, *g*_s_, and *I*WUE_i_ within and between leaves of an individual plant at any one time point (Fig. 8), as well as the change in parameter values with time and PPFD. At 200 µmol m^–2^ s^–1^ PPFD, ~70% of *A* values were between 5 µmol m^–2^ s^–1^ and 9 µmol m^–2^ s^–1^. When PPFD was increased to 800 µmol m^–2^ s^–1^, the median *A* value increased from 6.1 µmol m^–2^ s^–1^ to 10.3 µmol m^–2^ s^–1^ along with increased variation in the distribution of pixel values (1–30 µmol m^–2^ s^–1^; Supplementary Fig. S2a at *JXB* online). The variation in *A* and *g*_s_ resulted in heterogeneous patterns of *I*WUE_i_; for example, older leaves generally exhibited a 25% lower *I*WUE_i_ under 800 µmol m^–2^ s^–1^ than younger leaves (Fig. 9; Supplementary Fig. S2).

### Comparison of water use efficiency determined by imaging and gas exchange

A direct comparison between WUE_i_ determined from the combined imaging system and measurements taken using an IRGA (Fig. 9) showed a strong positive correlation. The measurements were taken under different environmental conditions including a range of PPFDs (200–800 µmol m^–2^ s^–1^) and a range of *C*_i_ values (35–1400 µmol mol^–1^) on different leaves from different *A. thaliana* plants. Measurements of WUE_i_ varied substantially with these different conditions, with values ranging from 0.008 µmol CO_2_/mmol H_2_O m^–2^ s^–1^ to 0.07 µmol CO_2_/mmol H_2_O m^–2^ s^–1^.

## Discussion

A new imaging system capable of near-instantaneous combined chlorophyll fluorescence and thermal imaging has been developed in order to generate images of *A*, *g*_s_, and *I*WUE_i_ from attached individual leaves and whole plants under strictly controlled environmental conditions. This is the first demonstration of the production of images of WUE_i_ and provides the prospect of rapid and non-destructive screening of plants for alterations in *A*, *g*_*s*_, and WUE_i_. Using this system, the impact of stomatal behaviour on *I*WUE_i_ was examined by comparing WT and known stomatal mutant *A. thaliana* plants and by monitoring plant responses to changing PPFD, as a driver of stomatal behaviour.

Values of *g*_s_ indicating the extent of stomatal opening (Omasa *et al.*, 1981; Jones, 1992) have previously been inferred from thermal images (Omasa *et al.*, 1981; Croxdale and Omasa, 1990; Inoue, 1990; Taconet *et al.*, 1995; Jones, 1999; Costa *et al.*, 2013); however, to date, only one study has produced images of *g*_s_ based on thermography. Omasa and Takayama (2003) analysed abscisic acid (ABA)-driven spatiotemporal changes in *g*_s_ in conjunction with fluorescence measurements of *F*_q_′/*F*_m_′ and non-photochemical quenching (NPQ) using a combined imaging system. The authors produced images of *g*_s_ from measurements of leaf temperature; however, these images only examined a small area of a single leaf (30×30mm). This present study also illustrated the potential for quantitative analysis of spatial and temporal variation in *g*_s_ and fluorescence in intact leaves that could provide valuable information regarding the coordination of stomatal and photosynthetic responses (Omasa and Takayama, 2003; West *et al.*, 2005; Aldea *et al.*, 2006; Chaerle *et al.*, 2007). Values of *g*_s_ estimated from thermal images have been found to be closely correlated with direct measurements of *g*_s_ from both porometry (Jones, 1999) and IRGA (Fig. 5) measurements, consequently giving a high degree of confidence in the images of *g*_s_ generated from this system. This has enabled spatial and temporal variation in *g*_s_ to be routinely monitored, allowing heterogeneity in *I*WUE_i_ to be assessed when *g*_s_ images are mapped to images of *A* (Fig. 3).

In ‘unstressed’ *A. thaliana* plants, spatial variation in *A*, *g*_s_, and *I*WUE_i_ was apparent within and between measurements of individual leaves, even under stable gas concentrations, PPFD, and temperature (Fig. 6, 8). However, once an intentional perturbation is introduced, such as the step-wise increase in PPFD, this variation in *I*WUE_i_ was greatly increased. These differences in magnitude and rate of change were mostly driven by *g*_s_. It is well established that stomatal responses are an order of magnitude slower than *A*, which often results in a disconnection between *A* and *g*_s_ following an alteration in the environment (Lawson *et al*., 2011, 2012), that manifests itself in spatial and temporal variation in *A* and *g*_s_ (Barradas and Jones, 1996; Weyers *et al.*, 1997; Lawson and Weyers, 1999; Lawson *et al.*, 2002). The degree of variation and the patterns observed are not surprising and are similar to many previous studies that have reported spatial and temporal variation in either *A* or *g*_s_ or other related variables (reviewed by Pospíšilová and Šantrůček, 1994; Weyers and Lawson, 1997; Lawson and Weyers, 1999) at scales that range from leaf to canopy (Weyers *et al.*, 1997) and in response to various abiotic and biotic stresses (Tang *et al.*, 2006; Ehlert and Hincha, 2008; Scholes and Rolfe, 2009; Bauriegel *et al.*, 2011; Nabity *et al.*, 2012). A lack of coordination between *A* and *g*_s_ and a lag in stomatal behaviour of between 5min and 10min is observed when *g*_s_ does not initially change as PPFD is increased, whereas *A* responds immediately to increasing PPFD (Fig. 7). The response of *A* generally occurs within 1min, while the response of *g*_s_ only occurs after a lag of several minutes and may take tens of minutes to complete (Meidner and Mansfield, 1968; Barradas and Jones, 1996; Wilmer and Fricker, 1996; Lawson and Weyers, 1999). It should however be noted that this is not the only explanation for variation in photosynthetic capacity (Miranda *et al.*, 1981); leaf anatomy (Sharkey, 1985; Terashima, 1992), leaf temperature (Hashimoto *et al.*, 1984), boundary layer thickness (van Gardingen and Grace, 1991), and water relations (Slavík, 1963) all possibly play a role. Such spatial and temporal heterogeneity accentuates the value and benefit of using an imaging approach to *A*, *g*_s_ and WUE_i_ over alternative traditional cuvette-based methods, which are generally confined to taking a single reading on an individual leaf or area of leaf which may or may not represent the average of the entire plant. The close relationship between WUE_i_ determined from images and traditional IRGA measurements (Fig. 9) illustrates the robustness of this imaging approach for rapidly assessing WUE_i_. Although not statistically significant, the imaged values in Fig. 9 tended to be in general lower than those measured directly. There are two plausible explanations for this; first, measurements were not taken on identical plants and, secondly, and more probably, these small differences are the result of a higher boundary layer conductance in the IRGA compared with the imaging system.

Although the potential to couple chlorophyll fluorescence and thermal imaging techniques has been explored in several studies (Omasa and Takayama, 2003; Chaerle *et al.*, 2007, 2009), most of these have been conducted at the small scale (Omasa and Takayama, 2003; Messinger *et al.*, 2006) and have focused on evaluating stomatal behaviour relative to photosynthetic performance (e.g. Chaerle *et al.*, 2005, 2007), including stomatal patchiness (Terashima *et al.*, 1988; Terashima, 1992; Beyschlag and Eckstein, 1998; West *et al.*, 2005), photoinhibition treatment (Badger *et al.*, 2009), and abiotic and biotic stress (Omasa and Takayama, 2003; Aldea *et al.*, 2006). Morison *et al.* (2008) were the first to highlight that the *F*_q_′/*F*_m_′ ratio from fluorescence, along with an indicator proportional to *g*_s_ (*I*_g_; see Jones, 1992) from thermography, provided the possibility to screen plant material non-destructively for *A* and the assimilation transpiration ratio (ATR; a parameter mathematically equivalent to WUE). Several previous studies have overlaid images of chlorophyll fluorescence and leaf temperature to detail the relationships between *A* and *g*_s_. However, crucially, this is the first study that has converted image data to values of *A* and *g*_s_ (using well-defined calibrations) and used these data to produce quantitative images of WUE_i_ collectively.

An important potential application of the imaging system is to screen plants for differences in WUE_i_. A demonstration of this potential is shown in Fig. 6 where clear differences in *I*WUE_i_ images between three WT *A. thaliana* and OST1-1 mutant plants were observed. Images of *A* and *g*_s_ from these plants indicate that differences in *g*_s_, rather than *A*, between the WT and the mutant are primarily responsible for the differences in *I*WUE_i_. As WUE_i_ is a function of both photosynthesis and stomatal behaviour, it is essential that future screening and selection of plants with improved WUE_i_ is not at the expense of overall carbon gain that may translate into reduced crop yield. The ability to determine whether differences in *A* or *g*_s_ account for differences in *I*WUE_i_ is essential for understanding the physiological mechanisms limiting WUE_i_, as high values of WUE_i_ can also be achieved with low *A* and *g*_s_, highlighting the importance of developing a system that can measure these two parameters independently. The effects of the differential contribution of *A* and *g*_s_ to WUE_i_ is exemplified in Fig. 6 showing a higher stomatal conductance in the OST mutants compared with the WT at 20min, but with no net gain in net CO_2_ assimilation, which resulted in a reduced WUE in these plants. Additionally, after 35min, *g*_s_ had increased in both the WT and mutant plants, although this increase was significantly greater in the WT plants, resulting in a greater *A*. The corresponding images of *I*WUE_i_ at this time point are in general lower than those observed 15min earlier, and the initial advantage observed in the WT plants has been lost although an overall greater carbon assimilation rate is apparent. These data provide a prime example of the importance of assessing the phenotypic components that drive WUE_i_ in screening approaches and protocols to ensure the appropriate combination of physiological traits is selected for improved WUE_i_. The ability to quantify *A* from images of *F*_q_′/*F*_m_′ is only possible due the built in capability of switching from atmospheric to low O_2_ in the measuring chamber which allows a linear relationship between *F*_q_′/*F*_m_′ and *A* to be observed, facilitating rapid comparative measurements of *A*, *g*_s_, and *I*WUE_i_. Six plants were imaged by the system in Fig. 6; however, it would be possible to image a greater number of smaller plants but with reduced pixel resolution. Lowering the O_2_ concentration to 20 mmol mol^–1^ has little effect on *g*_s_ over the short time periods required for the measurement of *F*_q_′/*F*_m_′, with stomatal responses only apparent after ~5–10min. However, it should be noted that determining WUE under non-photorespiratory conditions may differ if, for example, mesophyll conductance was different in one specimen relative to another under investigation.

Generally protocols for screening differences in *A* and *g*_s_ are made at steady state (Merlot *et al.*, 2002; Schurr *et al.*, 2006; Fiorani and Schurr, 2013); however the potential for plants to modify *A* and/or *g*_s_ in changing environments, such as those found in the field, may well be important in determining optimal productivity. The imaging system was designed to facilitate rapid changes in CO_2_ and O_2_ concentrations, humidity, PPFD, and temperature to generate dynamic responses of *A*, *g*_*s*_ and *I*WUE_i_ (Figs 7, 8). The success of this type of dynamic screening protocol depends entirely upon the ability to control the measurement conditions tightly, which does not usually take priority in many of the existing phenotypic platforms.

In conclusion, this novel imaging system provides the opportunity rapidly to assess spatial and dynamic differences in *A*, *g*_s_ and WUE_i_ in multiple plants under well-defined environmental conditions. This should facilitate improvements in the throughput of plants in phenotyping and screening protocols and consequently in the development of programmes for improved crop productivity.

## Supplementary data

Supplementary data are available at *JXB* online.

Figure S1. Pixel value distributions for images of CO_2_ assimilation (a and d), stomatal conductance (b and e), and *I*WUE_i_ (c and f) for a single WT (black) and OST mutant (grey) at 20min (*T*_20_) and at 35min (*T*_35_) under 200 µmol m^–2^ s^–1^ PPFD (see also Fig. 6).

Figure S2. Pixel value distributions for images of CO_2_ assimilation (a), stomatal conductance (b), and *I*WUE_i_ (c) for a single plant during a step-wise increase in PPFD (see also Figs 8 and 9).

Supplementary Data

## Abbreviations:

A: CO_2_ assimilation
C_a_: extracellular CO_2_ concentration
*C*_i_: intracellular CO_2_ concentration
*F*′: steady-state fluorescence measured under light
*F*_m_′: maximum fluorescence measured under light
*F*_q_′/*F*_m_′: photosystem II operating efficiency
*g*_c_: cuticle conductance
*g*_l_: leaf conductance to water vapour
*g*_s_: stomatal conductance
GUI: graphical user interface
*I*_g_: index of stomatal conductance
IRGA: infrared gas analysis
*I*WUE_i_: imaged intrinsic water use efficiency
PPFD: photosynthetic flux density
*r*_aw_: leaf boundary layer resistance to water vapour
*r*_HR_: parallel resistance to heat and radiative transfer
*r*_w_: leaf resistance to water vapour
s: slope of the curve relating saturation vapour pressure to temperature
*T*_Dry_: mean temperature of the dry standard
*T*_Leaf_: mean temperature of the leaf surface
*T*_Wet_: mean temperature of the wet standard
VPD: vapour pressure deficit
WUE: water use efficiency
WUE_i_: intrinsic WUE
γ: psychrometric constant
θ: temperature phrase normalizing leaf temperature with the wet and dry standards.
