# Supplementary material for: A novel system for spatial and temporal imaging of intrinsic plant water use efficiency
Source: J Exp Bot. 2013 Sep 16;64(16):4993–5007. doi: 10.1093/jxb/ert288 (PMC3830482; doi:10.1093/jxb/ert288)
Supplement: Supplementary Data [file supp_64_16_4993__index.html]

A novel system for spatial and temporal imaging of intrinsic plant water use efficiency — A novel system for spatial and temporal imaging of intrinsic plant water use efficiency — Supplementary Data 

# A novel system for spatial and temporal imaging of intrinsic plant water use efficiency

## Supplementary Data

Data files

**Files in this Data Supplement:**

- Supplementary Data - Supplementary Data
