# Supplementary material for: A novel system for spatial and temporal imaging of intrinsic plant water use efficiency
Source: J Exp Bot. 2013 Sep 16;64(16):4993–5007. doi: 10.1093/jxb/ert288 (PMC3830482; doi:10.1093/jxb/ert288)

A novel system for spatial and temporal imaging of intrinsic plant water use efficiency.

McAusland L, Davey PA, Kanwal N[1], Baker NR, Lawson T[*].

School of Biological Sciences, [1]School of Computing and Engineering Science, University of Essex, Wivenhoe Park, Colchester, Essex, CO4 3SQ, UK.

Supplementary Data

Figure S1: Pixel value distributions for images of CO_2 assimilation (a and d), stomatal conductance (b and e) and *i*WUE_i (c and f) for a single WT (black) and OST mutant (grey) at 20min (T_{20}) and at 35min (T_{35}) under 200 µmol m^{-2} s^{-1} PPFD (see also Figure 6). For ease of reading, every other x-axis value has been labelled.

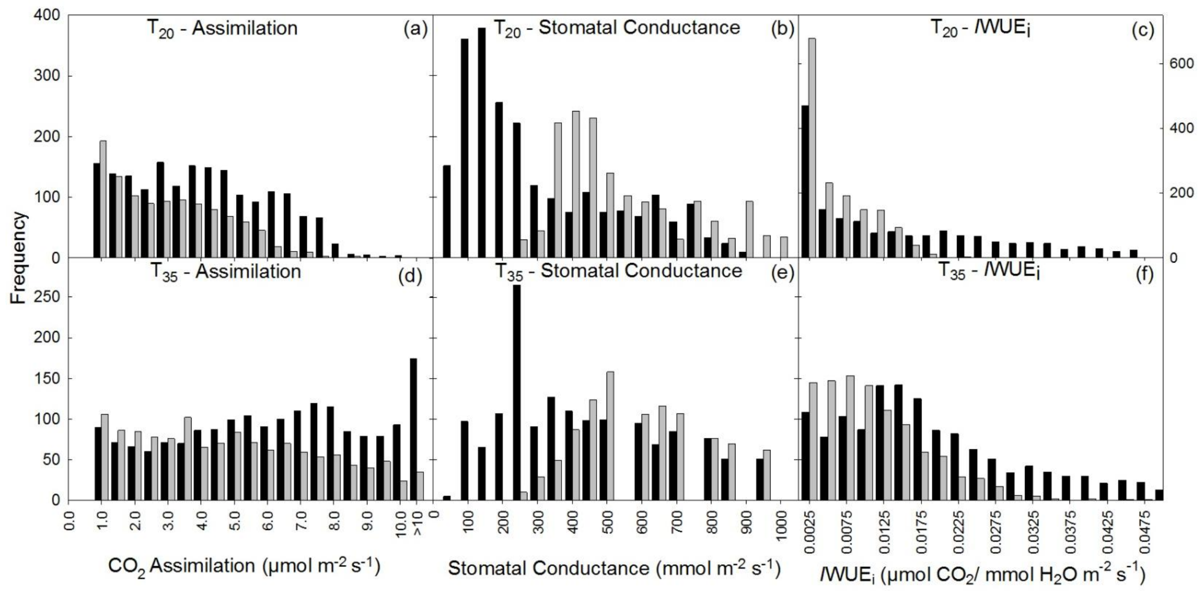

Figure S2: Pixel value distributions for images of CO_2 assimilation (a), stomatal conductance (b) and *I*WUE$_i$ (c) for a single plant during a step-wise increase in PPFD (see also Figures 8 and 9). Data from images of *A*, g_s and *I*WUE$_i$ were extracted after 9 minutes at 200 µmol m^{-2} (black) and after 30 minutes during 800 µmol m^{-2} s^{-1} PPFD (grey).

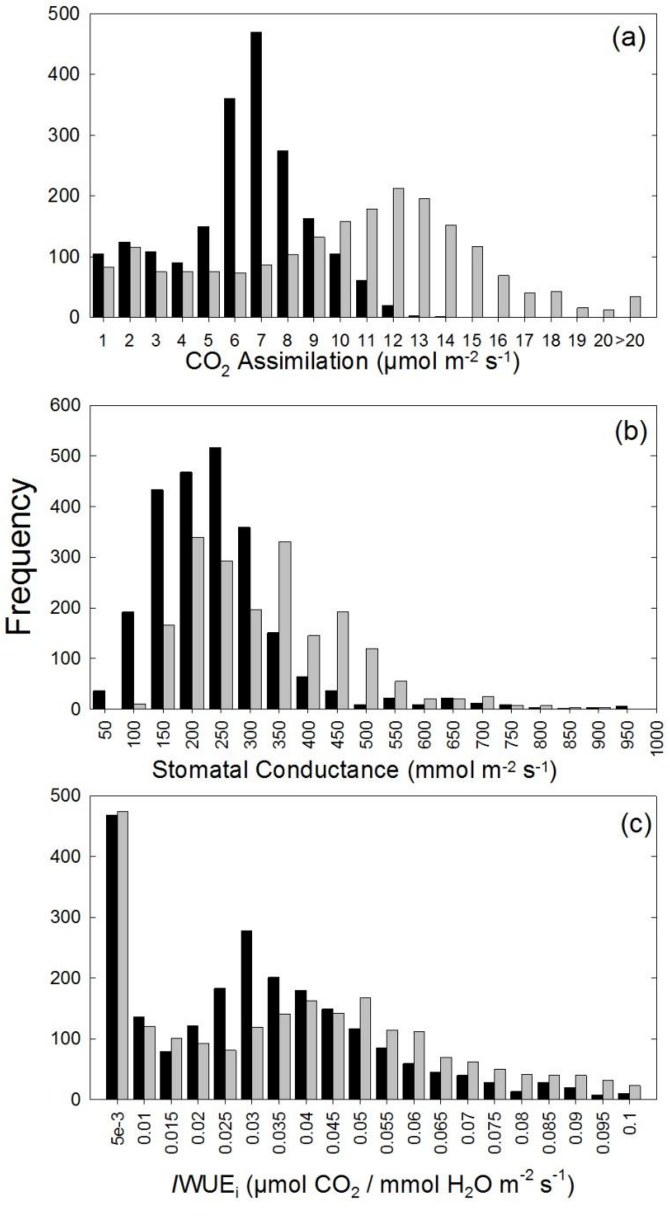

Supplement: Supplementary Data [file supp_ert288_jexbot102889_file001.pdf]
